# Antitumor immunity and immune-related toxicity during immune checkpoint inhibition: mechanistic overlap, tissue selectivity, and interventional evidence

**DOI:** 10.3389/fimmu.2026.1958441

**Published:** 2026-10-12

**Authors:** Chengyan Shi, Chunhai Li, Jun Yan

**Affiliations:** The Second Department of Oncology, The First Affiliated Hospital of Heilongjiang University of Chinese Medicine, Harbin, Heilongjiang, China

**Keywords:** antitumor immunity, autoimmunity, immune checkpoint inhibitor, immune-related adverse event, organ specificity, selective immunomodulation, tissue-resident memory T cell

## Abstract

By relaxing immune tolerance, immune checkpoint inhibitors can restore antitumor responses but can also cause immune-related adverse events across multiple organs. Efficacy and toxicity occur together in some patients, yet this association does not mean that tissue injury is an unavoidable cost of effective antitumor immunity. Shared antigens, lymphocyte clones expanded across tissues, B-cell activation, and weakened immune regulation create overlap between tumor control and normal-tissue injury during immune priming and effector responses. Whether toxicity persists in a particular organ may be shaped by local antigen presentation, tissue residency, cellular interactions, epithelial barrier disruption, and amplification of innate inflammation, but direct support for these processes is strongest in colitis and remains less complete across other organs. Because residency programs also support tumor-reactive T cells, localization within an organ does not automatically preserve antitumor activity. Restricting organ homing, protecting tissue barriers, and targeting pathways such as IL-5, IL-6, JAK, and NLRP3 have shown potential to reduce toxicity in case series or animal models, but their effects on long-term tumor control remain insufficiently evaluated. Determining whether a treatment controls toxicity while preserving antitumor activity requires concurrent assessment, within the same cohort, of objective organ response, glucocorticoid exposure, resumption of immunotherapy, duration of tumor response, and survival. Current evidence supports further study of mechanisms that sustain toxicity within affected organs, but it is not yet sufficient to establish that most candidate strategies achieve durable, clinically meaningful separation of toxicity from antitumor benefit.

## 1 Introduction

Immune checkpoint inhibitors (ICIs) enhance antitumor T-cell responses by blocking inhibitory signals such as CTLA-4, PD-1, or PD-L1. The same treatments can disrupt peripheral tolerance and cause immune-related adverse events (irAEs) involving the skin, gastrointestinal tract, liver, lungs, endocrine glands, neuromuscular system, and heart. Current guidelines stratify recommendations for ICI interruption, glucocorticoids, and subsequent immunosuppression by the affected organ and severity, although many recommendations still rely largely on expert consensus and nonrandomized evidence (1).

The association between irAEs and tumor outcomes is not uniform. An analysis of 9521 patients from 14 atezolizumab trials found that grade 1–2 irAEs were associated with longer overall survival, whereas grade 3–4 irAEs did not show the same overall benefit. Severe pneumonitis and colitis were associated with poorer survival, and the direction of the association varied by organ and severity (2). Thus, irAEs are not a uniform surrogate for efficacy, and whether toxicity control alters antitumor immunity must be evaluated directly in treatment studies.

Mechanistic studies suggest that tumors and affected organs may share antigens or T-cell clones, but organ injury also depends on local resident cells, spatial cellular proximity, tissue barriers, and myeloid inflammation. Some processes contribute to both tumor control and tissue injury, whereas others may become more important after immune cells enter a particular organ. Systemic immune suppression, modulation of tissue recruitment, interruption of local effector pathways, and barrier protection therefore cannot be assumed to have equivalent effects on antitumor immunity. The most direct tissue-mechanistic and interventional data currently come from colitis and myocarditis. Observations from skin, lung, liver, kidney, endocrine, neurologic, eosinophilic, and transplant-associated toxicities constitute organ-specific evidence rather than interchangeable support for a universal model (**Figure 1**).

**Figure 1:**
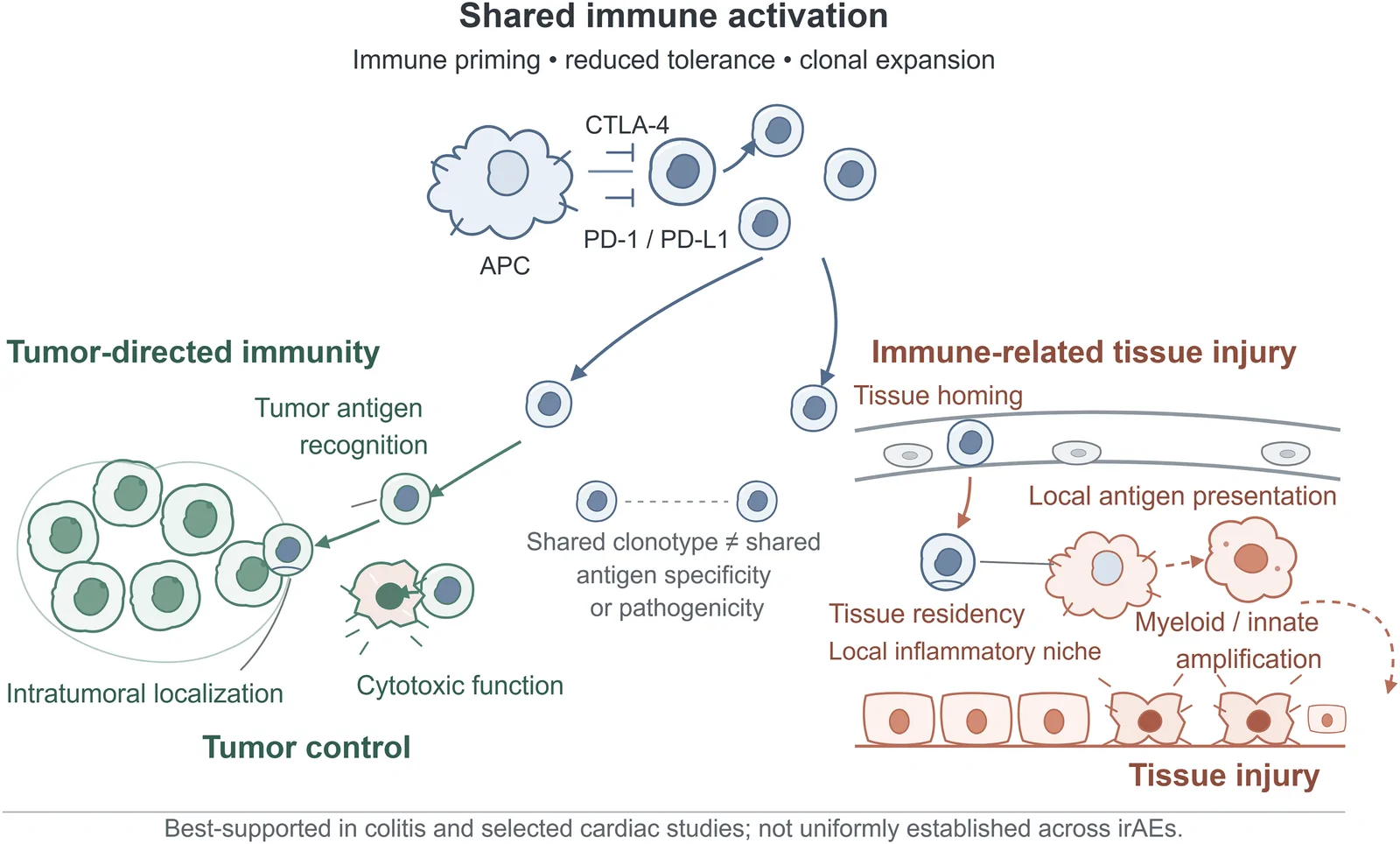
Shared immune activation and divergence into tumor-directed immunity and immune-related tissue injury during immune checkpoint inhibition. Checkpoint blockade promotes immune priming, reduces peripheral tolerance, and expands lymphocyte clones that may contribute to both tumor-directed immunity and normal-tissue injury. In the tumor branch, antitumor activity depends on tumor-antigen recognition, intratumoral localization, and cytotoxic function. In affected tissues, recruitment is followed by local antigen presentation, tissue residency, inflammatory cellular interactions, and myeloid or innate inflammatory amplification that may contribute to tissue injury and persistence. These processes may overlap in time and are not uniformly established across organs. Detection of shared T-cell clonotypes across tumors and affected tissues establishes sequence overlap but does not, by itself, demonstrate shared antigen specificity or pathogenicity. Evidence for the depicted local persistence mechanisms is strongest in colitis and selected cardiac studies and should not be generalized uniformly across irAEs.

Here, selective immunomodulation denotes a clinically testable treatment property rather than a presumed feature of a tissue-restricted or downstream target. Mechanistic or anatomical restriction provides a rationale for selectivity, but is insufficient to infer tumor sparing because tumor-cell JAK–IFN-γ integrity, tissue-residency programs, and IL-6-linked inflammatory circuits can also shape tumor immunity and responses to checkpoint blockade (3–5). A clinically credible claim therefore requires, in the same treated cohort, meaningful toxicity improvement—preferably an objective organ response with reduced glucocorticoid or additional immunosuppressive exposure and ICI continuation or resumption when appropriate—together with longitudinal oncologic assessment, including response durability, subsequent progression, progression-free survival, and overall survival.

The effort to separate graft-versus-host disease (GVHD) from graft-versus-leukemia (GVL) provides a useful conceptual precedent. In allogeneic hematopoietic cell transplantation, tissue-directed trafficking, local treatment, and tissue-tolerance strategies are being studied to limit normal-tissue injury while retaining immune activity in the bone marrow, but reliable clinical separation has not been established (6). The analogy is incomplete: GVHD/GVL is driven by donor alloreactivity in a transplant setting, whereas ICI therapy reinvigorates endogenous immunity against solid tumors. GVHD/GVL provides precedent for testing tissue-directed approaches, but it does not establish that analogous strategies during ICI treatment will preserve antitumor immunity. Preservation of antitumor control must still be demonstrated directly.

## 2 Shared antigens, clonal expansion, and T–B-cell cooperation create partial mechanistic overlap

### 2.1 Shared antigens and T-cell clones in tumors and affected tissues

When tumors and normal tissues express identical or related antigens, ICI-expanded T cells may recognize both. In a prospective cohort of patients with non-small-cell lung cancer treated with anti-PD-1 therapy, 25 of 73 patients developed autoimmune skin toxicity. Shared antigens were identified in tumors and affected skin, and the corresponding antigens stimulated CD8 or CD4 T cells *in vitro*. These findings support a possible role for shared antigens in skin toxicity, but they arose from one tumor type and one affected organ and cannot explain other irAEs (7).

In 2 patients who developed fatal myocarditis, myositis, and rhabdomyolysis after ipilimumab plus nivolumab, high-frequency T-cell clones in the myocardium were also found in the tumor and skeletal muscle. The tumors expressed muscle-associated antigens, providing a candidate explanation for attack on muscle tissue by tumor-activated clones (8). The presence of identical T-cell receptor (TCR) sequences across tissues nevertheless has several possible explanations: a clone may recognize the same antigen in both sites, cross-react with related antigens, or simply be detected in both samples because of sampling and expansion biases affecting a high-frequency clone.

TCR-sequencing studies show that estimates of shared clonotypes are influenced by sample size, tissue compartment, sampling time, and batch. Evidence for pathogenicity therefore progresses beyond sequence overlap in several steps. Clonal overlap establishes only sequence identity. Identification of a candidate shared or cross-reactive antigen narrows the mechanism, and functional stimulation of the relevant clone establishes antigen responsiveness. Spatial localization beside injured target cells and a quantitative association with tissue damage strengthen biological relevance, but causal support requires perturbation showing that removal or blockade of the clone or pathway reduces injury while appropriate tumor and unaffected-tissue controls exclude bystander expansion (7–9). Few human irAE studies traverse this sequence from clonotype to causal tissue injury. Until the pathogenic clones and their antigens are defined, the consequences of removing a particular T-cell population for tumor control will remain difficult to predict.

The same clone may also acquire different functions after entering different tissues. Local antigen presentation, TGF-β and IL-15 signaling, inhibitory receptors, cell adhesion, oxygen availability, and nutrient conditions all influence T-cell residency, survival, and effector state (4). Resident T cells within tumors can contribute to treatment responses, while resident cells can be reactivated in cutaneous and gastrointestinal irAEs. Blocking tissue entry or maintenance of residency could therefore reduce toxicity but also deplete intratumoral effector cells. Downstream signals that predominate in the affected organ may provide more anatomically restricted targets than the resident phenotype itself, but their effects on antitumor immunity still require direct assessment.

### 2.2 B-cell activation, Tph-like help, and regulatory T-cell restraint

Peripheral B-cell changes can emerge during the first treatment cycle after combined CTLA-4 and PD-1 blockade. These changes include a reduction in total B cells, increases in CD21^low B cells and plasmablasts, and expansion of selected clones. They precede clinical irAEs and are associated with the frequency and timing of subsequent high-grade irAEs (10). Peripheral B-cell changes may therefore help identify patients at increased risk, but the available observational associations do not show whether preventive B-cell-directed intervention is beneficial.

Patient samples, coculture experiments, and humanized mouse models further showed that combined checkpoint blockade expanded an ICOS-positive peripheral helper T-cell-like population and promoted responses involving CD21^low CD11c-positive B cells and plasmablasts. ICOS-positive CD4 T cells enhanced plasmablast differentiation and IgG production, and this response increased after regulatory T-cell depletion. Among patients who did not develop high-grade irAEs, a higher proportion of ICOS-positive CD4 T cells acquired a regulatory phenotype after treatment (11). The balance between effector T–B-cell cooperation and regulatory restraint may track toxicity risk more closely than expansion of either cell population alone.

ICOS-positive T cells and B-cell responses may also contribute to antitumor immunity, making wholesale depletion of these populations unlikely to be selective. The available data are better suited to early risk identification and toxicity phenotyping than to direct therapeutic targeting. Blocking a specific helper signal or augmenting regulatory T cells would have a clearer mechanistic basis only if humoral immunity or T–B-cell interactions were shown to dominate a defined toxicity subtype.

### 2.3 TREX1 deficiency links autoimmune susceptibility and antitumor responses to shared immune programs

Genetic studies in mice showed that persistent loss of the cytosolic DNA exonuclease TREX1 caused multiorgan CD8 T-cell infiltration, myocarditis, and Sjögren-like manifestations while enhancing CD8 T-cell-driven antitumor immunity. By contrast, inducible deletion in adulthood was better tolerated and retained antitumor activity (12). Innate DNA sensing and adaptive T-cell effector responses can therefore influence both autoimmune susceptibility and tumor control, while the timing of intervention and host state alter the resulting phenotype.

This mouse model illustrates how host background and the timing of immune activation can shift the balance between self-tissue injury and tumor control. It is not equivalent to patient-derived irAE evidence, but it supports distinguishing the generation of effective antitumor clones from the persistence of injury in nonmalignant organs. The latter also depends on survival, effector, and tissue-damaging signals acquired after cells enter the affected organ.

## 3 Resident immunity, barrier disruption, and innate inflammation in the colon and heart

### 3.1 IFN-γ-producing CD8 tissue-resident memory T cells in ICI colitis

Single-cell analysis of ICI colitis identified expanded cytotoxic and proliferative CD8 T-cell states, with TCR evidence that a substantial fraction of colitis-associated cells arose from tissue-resident populations (13). In an independent study, multiparameter flow cytometry, tissue imaging, and transcriptomic analyses comparing ICI colitis, ICI-treated patients without colitis, ulcerative colitis, and healthy controls identified IFN-γ-producing CD8 tissue-resident memory T (TRM) cells as a prominent activated population. Their activation correlated with clinical and endoscopic severity, and ICI colitis differed from ulcerative colitis in immune and epithelial signaling. On the basis of this pathway, 1 patient with refractory disease was treated with the JAK inhibitor tofacitinib and achieved remission, but a single therapeutic case cannot establish general efficacy or tumor safety (14).

Gut entry and mucosal persistence are related but distinct processes: α4β7 and CCR9 contribute to intestinal homing, whereas local cytokines and adhesion programs support retention and tissue adaptation (15). TRM cells can rapidly release effector molecules after local stimulation. Systemic exposure to an ICI therefore need not produce uniform tissue injury; pre-existing resident cells, chemotactic signals, and the local stromal state may jointly determine whether persistent inflammation develops in the intestine (13, 14). Tumor-reactive T cells can also adopt residency programs (4), so TRM cells are not intrinsically safe therapeutic targets. Cellular interactions, downstream cytokines, or barrier responses that predominate in the affected organ may provide more anatomically restricted targets, although anatomical restriction alone does not show that antitumor immunity will be preserved.

### 3.2 ICI-bound T cells form distinct cellular niches in colonic tissue

Tracking of ICI-bound T cells, single-cell sequencing, and subcellular-resolution spatial transcriptomics in blood and intestinal tissue from patients with ICI colitis identified ICI-bound Tfh, Tph, Th17, Treg, and HAVCR2-positive CD8 T cells. These cells were enriched in distinct niches within colitic tissue, and niche composition also distinguished ICI colitis from ulcerative colitis (16).

Because checkpoint inhibitors bound multiple effector, helper, and regulatory T-cell populations, drug binding itself did not show that the bound cells were pathogenic. Spatial proximity can reveal whether T cells form local inflammatory units with epithelial, myeloid, or B cells, but colocalization alone does not identify which population sustains tissue injury (16). Genetic, pharmacologic, or ex vivo perturbation is needed to separate pathogenic circuits from changes secondary to inflammation.

### 3.3 MLCK1-dependent tight-junction leak amplifies intestinal injury

Studies using human ICI colitis samples, organoids, and tumor-bearing mice showed that T-cell-derived tumor necrosis factor activated an MLCK1-dependent tight-junction leak pathway and disrupted the intestinal epithelial barrier. Genetic deletion of MLCK or pharmacologic disruption of the MLCK1–FKBP8 interaction with epicatechin reduced colitis and barrier injury in model systems without attenuating the antitumor effect of ICIs (17).

This study localized a potentially tractable process to the epithelial response to inflammatory signaling rather than to direct suppression of antitumor T cells. Epicatechin was used mainly as a protective intervention concurrent with ICI treatment, and tumor outcomes were based primarily on short-term volume and terminal weight. The experiments therefore did not establish whether existing colitis could be reversed or whether long-term tumor control would be preserved. Effective exposure, target specificity, pharmacokinetics, and clinical safety also remain unconfirmed in patients with ICI colitis, leaving barrier protection as a preclinical candidate strategy.

### 3.4 The NLRP3 inflammasome in ICI-associated cardiac injury

In models of ICI-associated cardiac injury, the NLRP3 inflammasome inhibitor MCC950 reduced the infiltration of pathogenic macrophages and cytotoxic and activated T cells and improved cardiac function. In a concurrent-treatment experiment using B16F10 tumor-bearing mice, reduced cardiac injury was accompanied by lower tumor volume and weight (18). Publicly available peripheral-blood single-cell data also showed increased NLRP3 and IL1B expression in monocytes from patients with ICI myocarditis, although this does not substitute for functional validation in human myocardium.

The investigators separately initiated MCC950 after cardiac injury had developed; tumors were not implanted in this experiment because of animal-welfare constraints. The first experiment provides a joint cardiac–tumor signal during preventive or concurrent intervention, whereas the second addresses only whether established cardiac injury can be improved. These experiments cannot be combined as evidence that treatment of established myocarditis preserves tumor control (18) (**Table 1**).

**Table 1:** Mechanistic processes linking antitumor immunity and immune-related toxicity: overlap, tissue restriction, and therapeutic implications.

Mechanism	Principal cells or pathway	Antitumor role	Role in irAEs	Evidence	Basis for organ restriction	Therapeutic implication	Key boundary
Checkpoint release and tolerance disruption	CTLA-4, PD-1, or PD-L1 blockade; effector T cells	Restores antitumor T-cell responses	Reduces peripheral tolerance	Human clinical and mechanistic studies (1, 2)	Tolerance and local antigen presentation vary by organ	Dose, timing, and sequence may alter efficacy and toxicity	Shared upstream process with little intrinsic organ selectivity; efficacy associations vary by organ and grade (2).
Shared or cross-reactive antigens	Tumor and normal-tissue antigens	Supports tumor recognition	May direct expanded clones toward antigen-bearing tissue	NSCLC skin-toxicity cohort with *in vitro* stimulation (7)	Injury requires antigen presentation in the organ	May support antigen-informed risk assessment	One tumor type and organ; shared antigen does not identify a tumor-sparing target.
Shared TCR clonotypes across tumor and tissue	High-frequency T-cell clones in tumor and affected organ	May mark tumor-directed expansion	Raises shared-recognition or cross-reactivity hypotheses	Two fatal myocarditis cases; TCR-sequencing studies (8) (9)	Distribution may reflect true recognition, expansion, or sampling	Pair clone tracking with antigen and functional assays	Sequence identity does not establish antigen specificity or tissue pathogenicity.
Tph-like T-B-cell cooperation and plasmablast responses	ICOS-positive Tph-like cells; CD21low CD11c-positive B cells; plasmablasts	May contribute to tumor immunity	Early expansion tracked high-grade irAEs	Patient samples, coculture, and humanized mice (10, 11)	Helper signals may dominate selected toxicity phenotypes	More useful for risk phenotyping than wholesale depletion	Broad T- or B-cell depletion is unlikely to be selective.
Regulatory T-cell restraint	Treg cells; ICOS-positive CD4 regulatory phenotype	Can constrain antitumor responses	Loss of restraint permits effector T-B cooperation	Patient samples and functional experiments (11)	Effector-regulatory balance may vary by phenotype	Requires toxicity-specific targeting and tumor assessment	Cell counts or spatial enrichment do not prove pathogenic necessity.
TREX1-related innate-adaptive convergence	TREX1; innate DNA sensing; CD8 T cells	Persistent loss enhanced CD8-driven tumor control	Caused multiorgan CD8 infiltration and autoimmunity in mice	Persistent versus inducible mouse deletion (12)	Host state and timing altered the phenotype	Separates clone generation from persistence of organ injury	Mouse genetics do not constitute patient irAE evidence.
Tissue homing and trafficking	α4β7, CCR9, and chemotactic signals	Homing programs also direct tumor-reactive cells	Directs lymphocytes toward affected organs	Gut-homing biology and human vedolizumab studies (15) (24, 25)	Receptor-ligand pairs can be organ enriched	Restrict organ entry in defined settings	Preventive benefit and durable tumor preservation remain unproven.
Tissue residency and TRM programs	IFN-γ-producing CD8 TRM and related resident states	Intratumoral resident cells can support response	Resident cells can reactivate in epithelial irAEs	Human colitis and ICI-TRM studies (4, 13, 14)	Local retention and survival support persistence	Target downstream circuits rather than residency alone	Residency supports both tumor control and tissue injury.
Local inflammatory cellular niches	Tfh, Tph, Th17, Treg, HAVCR2-positive CD8, epithelial, myeloid, and B cells	Local interactions may support tumor effectors	Distinct colonic niches accompany inflammation	Human single-cell and spatial profiling (16)	Niche composition is tissue specific	Perturb candidate local interactions	Colocalization does not establish causal maintenance.
Epithelial barrier disruption/MLCK1-dependent leak	Tight junctions; MLCK1-FKBP8; TNF	Barrier protection avoids direct T-cell suppression	TNF-driven leak amplifies colitis	Human tissue, organoids, and tumor-bearing mice (17)	Intestinal epithelial response	Preclinical barrier protection	Concurrent protection does not prove reversal of established colitis or durable tumor safety.
Innate inflammatory amplification/NLRP3 and myeloid circuits	NLRP3; macrophages; IL-1β; neutrophils	Myeloid circuits shape tumor immunity	Macrophage-inflammasome activity amplified cardiac injury in models	Cardiac-injury mice and patient blood single-cell data (18)	Local cardiac inflammatory context	Preclinical NLRP3 inhibition	Concurrent tumor data and established-injury data came from separate experiments.
Phenotype-specific cytokine dependence (IL-5 axis)	IL-5-dependent eosinophils	Effect on tumor immunity remains uncertain	Supports eosinophil-dominant toxicity	Three-patient case series (26)	Defined eosinophilic phenotype	Phenotype-matched IL-5-axis blockade	Organ-specific cases with limited tumor follow-up.
Phenotype-specific cytokine dependence (IL-6)	IL-6 and myeloid inflammation	Context-dependent effects on tumor immunity	May sustain selected established irAEs	Melanoma cohort and animal models (5)	Pathway activity may define a treatment subgroup	Candidate treatment for established toxicity	Uncontrolled human data do not establish response durability.
Phenotype-specific cytokine dependence (IFN-γ/JAK-related)	IFN-γ and JAK-dependent TRM circuits	JAK-IFN-γ integrity can support checkpoint response	Contributes to colitic inflammation	Tumor-response mechanistic evidence and human colitis mechanism/treatment evidence (3, 14)	Strongest evidence is in colitis	Hypothesis-generating rescue option	Single case cannot establish efficacy or tumor safety.

This table summarizes selected mechanistic processes and does not imply equivalent cross-organ support. Colitis includes convergent human tissue and perturbation studies, whereas most other rows derive from organ-specific cohorts, case series, or models that answer different questions.

ICI, immune checkpoint inhibitor; irAE, immune-related adverse event; MLCK1, myosin light-chain kinase 1; NSCLC, non-small-cell lung cancer; TCR, T-cell receptor; Tfh, T follicular helper; Tph, T peripheral helper; Treg, regulatory T cell; TRM, tissue-resident memory T cell.

### 3.5 Evidence for local persistence differs across irAE organs

The colon currently provides the most integrated evidence for local persistence because patient tissue profiling is linked to spatial cellular niches and, for epithelial barrier injury, to organoid and animal perturbation (14, 16, 17). Cardiac evidence is less integrated: fatal human cases identify candidate clones, while NLRP3 inhibition has been tested mainly in mouse models (8, 18). Recruitment and local inflammatory amplification recur in studies of several organs, but the responsible cells and pathways differ, and no common causal maintenance mechanism has been demonstrated across irAEs.

Outside the colon and heart, human studies usually identify local cell states rather than causal maintenance nodes. Single-cell and TCR analysis of bronchoalveolar lavage fluid from 11 patients with ICI pneumonitis and 6 cancer controls found enrichment of T-helper 17.1 cells and pro-inflammatory monocytes, but their proposed feedforward interaction was not perturbed in that study (19). In checkpoint inhibitor-related hepatitis, profiling of 22 patients with hepatitis and 7 ICI-treated controls, with liver immunostaining in 4 cases, identified activated monocytes, cytotoxic CD8 T cells, and intrahepatic colocalization of these populations (20). A pilot nephritis study detected urine T cells and kidney-enriched TCR sequences in three sequenced cases, but lacked tumor tissue, antigen-specificity testing, and causal perturbation (21).

Endocrine and neurologic toxicities also do not fit a single reversible local-inflammation model. ICI-related thyroid dysfunction may evolve from destructive thyroiditis to persistent hypothyroidism, whereas ICI-associated diabetes generally reflects permanent β-cell loss; long-term management therefore often depends on hormone replacement rather than continued suppression of an active inflammatory circuit (22). In a series of 63 patients with neurologic autoimmunity, manifestations included central nervous system, neuromuscular, combined, and retinal syndromes, with heterogeneous neural autoantibody profiles (23). Across organs, the recurring observation is that local cells and tissue state modify toxicity; the specific cells, injury process, reversibility, and treatment target remain organ dependent.

Timing also limits mechanistic interpretation. Peripheral immune changes detected before symptoms may reflect susceptibility or initiation (10), whereas tissue and fluid samples obtained after the onset of colitis, pneumonitis, hepatitis, or nephritis identify cell states present during established injury (14, 19–21). Without serial sampling across onset and recovery, these observations leave unresolved whether recruitment, tissue residency, barrier disruption, or myeloid activation initiates toxicity, maintains established injury, or changes in role over time.

## 4 Human and preclinical evidence for mechanistically restricted interventions

The biological question changes with intervention timing. Treatment given before or concurrently with ICI exposure tests prevention; treatment at symptom or biomarker onset tests early interruption; treatment after objective organ injury tests reversibility; and rescue treatment during severe or life-threatening dysfunction tests immediate organ preservation and survival. Evidence from one setting cannot substitute for another. The human studies summarized below are concentrated in established or refractory toxicity and rescue treatment; prospectively defined early-treatment windows remain insufficiently studied.

### 4.1 Vedolizumab restricts lymphocyte homing to the gut

Vedolizumab, an antibody against α4β7 integrin, primarily restricts lymphocyte homing to the intestine. In a case series of 7 patients with glucocorticoid-dependent or partly refractory ICI enterocolitis, all except 1 achieved glucocorticoid-free remission after vedolizumab, with normalization of fecal calprotectin and no clearly treatment-related adverse events (24).

This small series suggests that intestinal toxicity can be controlled without directly depleting systemic T cells, but it did not include a systematic comparator for tumor outcomes. Preventive use has also not shown a clear advantage. A nonrandomized phase Ib study of vedolizumab with nivolumab and ipilimumab enrolled only 12 patients with advanced melanoma and was closed early after two patients developed grade 3 diarrhea or colitis, meeting a prespecified stopping rule. The sample was also too small to assess antitumor efficacy (25). The biological rationale for restricting gut homing has therefore not translated into a demonstrated preventive benefit.

### 4.2 IL-5- and IL-6-directed interventions require matching toxicity phenotypes

Eosinophilia may accompany ICI efficacy but can also injure the lungs, fascia, skin, or other organs. In three patients with eosinophilic irAEs that were glucocorticoid-refractory or persisted after ICI discontinuation, mepolizumab or benralizumab produced rapid improvement in symptoms and peripheral eosinophil counts, without a clear signal of tumor progression during follow-up (26). The number of cases and duration of oncologic follow-up were limited. These observations support further study of IL-5-axis inhibition in eosinophil-dominant toxicity but do not exclude an effect on antitumor immunity.

IL-6, neutrophils, and chemotaxis-related markers were increased in tissue from patients with ICI colitis. In B16.BL6 melanoma and CT26 colon cancer models, IL-6 blockade attenuated experimental autoimmune encephalomyelitis and improved some tumor outcomes (5). In most of these experiments, treatment was administered early and concurrently with tumor implantation, ICI therapy, and autoimmune induction, thereby addressing prevention or early intervention rather than treatment of established toxicity. Experimental autoimmune encephalomyelitis also does not represent common organ-specific irAEs.

The same study retrospectively evaluated 31 patients with melanoma and irAEs who received tocilizumab or sarilumab. A total of 74% improved after a median of 2 months. Among 26 evaluable patients, the best overall response rates to ICI before and after IL-6 blockade were 57.7% and 65.4%, respectively, with no statistically significant difference (5). Patient selection, toxicity type, and prior immunosuppression were heterogeneous, and there was no control group. A best response already achieved does not disappear immediately after subsequent immunosuppression; this before-and-after comparison therefore did not establish how long responses persisted or whether the subsequent risk of progression changed.

### 4.3 Multicomponent rescue therapy for severe myocarditis prioritizes immediate mortality risk

In a sequential cohort of 40 patients with definite ICI myocarditis, myotoxicity-related mortality was 60% among the first 10 patients treated according to the previous pathway. Among the subsequent 30 patients, systematic screening for respiratory muscle involvement, proactive ventilatory support, high-dose abatacept adjusted according to CD86 receptor occupancy, and concomitant ruxolitinib were followed by one death. The investigators characterized these findings as hypothesis-generating evidence (27).

The before-and-after design, intensified respiratory support, multicomponent therapy, and changes in case recognition could all have influenced mortality, preventing attribution of the difference to either abatacept or ruxolitinib alone. CD86 receptor occupancy reflects the pharmacodynamic intensity of abatacept but does not identify the mechanism driving myocardial injury. Life-threatening myocarditis first requires prevention of irreversible organ damage. Mechanistic testing can proceed alongside rescue treatment only if it does not delay ventilation, circulatory support, or adequate immunosuppression.

One kidney transplant recipient with melanoma developed graft rejection and colitis after PD-1 blockade. The addition of sirolimus was followed by graft tolerance and maintained antitumor activity; cytotoxic CD8 T cells and eosinophils decreased, regulatory T cells were maintained, and IFN-γ-positive CD4 T-cell responses were preserved (28). This unusual setting suggests that mTOR modulation may alter the relative contributions of different immune populations, but a single case, the transplant context, and multiple concomitant treatments preclude extrapolation to the broader ICI-treated population (**Figure 2**; **Table 2**).

**Figure 2:**
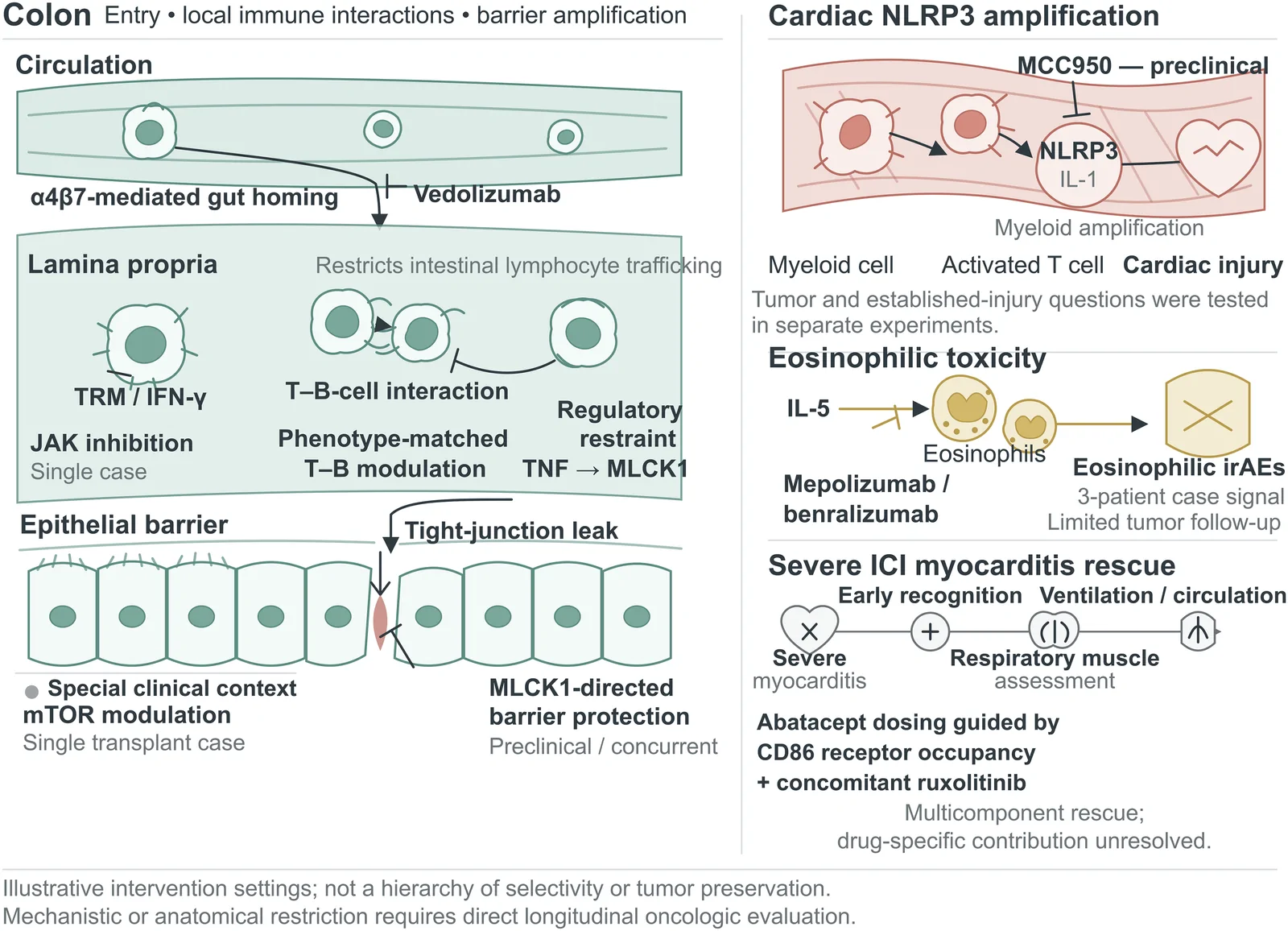
Organ- and phenotype-specific mechanisms and potential intervention points for immune checkpoint inhibitor-related toxicity. Selected interventions are shown within the organ or clinical contexts in which the supporting evidence was generated rather than as a hierarchy of selectivity. In the colon, vedolizumab restricts α4β7-mediated lymphocyte trafficking, JAK inhibition has a single-case treatment signal in refractory colitis, and MLCK1-directed barrier protection targets TNF-associated tight-junction leak primarily in preclinical concurrent-treatment models. T-cell–B-cell interactions and regulatory restraint identify local adaptive processes that may support phenotype-matched intervention, whereas mTOR modulation is shown separately because the available clinical evidence derives from a single transplant-associated case. In cardiac models, MCC950 targets NLRP3-associated myeloid inflammatory amplification; tumor-bearing and established-injury experiments addressed separate questions and should not be combined as evidence of tumor preservation during treatment of established cardiac injury. IL-5-axis blockade has been reported in a three-patient series of eosinophilic irAEs with limited oncologic follow-up. Severe ICI myocarditis is shown as a multicomponent rescue setting in which early recognition, respiratory-muscle assessment, ventilatory or circulatory support, pharmacodynamically guided abatacept dosing, and concomitant ruxolitinib were implemented together. These examples are illustrative and do not constitute a hierarchy of selectivity or tumor preservation. Mechanistic or anatomical restriction requires direct longitudinal oncologic evaluation. CD86, cluster of differentiation 86; ICI, immune checkpoint inhibitor; IL, interleukin; irAE, immune-related adverse event; JAK, Janus kinase; MLCK1, myosin light-chain kinase 1; mTOR, mechanistic target of rapamycin; NLRP3, NOD-, LRR- and pyrin domain-containing protein 3; TRM, tissue-resident memory T cell; TNF, tumor necrosis factor.

**Table 2:** Representative interventions for immune-related adverse events and available evidence regarding antitumor outcomes.

Intervention	Target	irAE setting	Timing	Evidence and sample	Toxicity result	Tumor assessment	Key limitation	Interpretation
Gastrointestinal toxicity								
Vedolizumab	α4β7; gut homing	ICI enterocolitis	Established toxicity; prevention/concurrent study	7-patient case series; 12-patient nonrandomized phase Ib study (24, 25)	Most treated cases achieved steroid-free remission; preventive study stopped after two grade 3 events	Direct but insufficient in the preventive cohort; not systematic in the treatment series	Small samples; no tumor comparator; preventive benefit unproven	Established-treatment signal; prevention and durable tumor preservation unproven.
Tofacitinib	JAK-related TRM/IFN-γ signaling	Refractory ICI colitis	Established refractory toxicity	Single patient (14)	Remission reported	Not assessed	Single case	Hypothesis-generating only.
MLCK1 targeting/epicatechin	MLCK1-FKBP8; epithelial barrier	ICI colitis models	Prevention/concurrent treatment	Human tissue, organoids, and tumor-bearing mice (17)	Reduced barrier injury and colitis in models	Direct but limited to short-term tumor volume and terminal weight	No established-colitis treatment or long-term tumor data	Preclinical prevention candidate.
Cardiac toxicity								
MCC950	NLRP3 inflammasome	ICI cardiac-injury models	Concurrent tumor experiment; separate established-injury experiment	Mouse models; patient blood single-cell data (18)	Reduced immune infiltration and improved cardiac function	Direct but short-term with concurrent dosing; absent from established-injury experiment	Tumor and established-injury questions were tested separately	Preclinical signal; established-treatment tumor preservation unproven.
Abatacept + ruxolitinib + intensive supportive care	Costimulatory/JAK blockade plus organ support	Severe ICI myocarditis	Life-threatening rescue	Sequential cohort, 40 patients (27)	Myotoxicity mortality fell from 60% to 1/30	Not adequately assessed	Multiple care components changed simultaneously	Important rescue signal; drug-specific effect unresolved.
Phenotype-matched cytokine strategies								
Mepolizumab/Benralizumab	IL-5 or IL-5 receptor	Eosinophilic irAEs	Established refractory or persistent toxicity	Three patients (26)	Symptoms and eosinophil counts improved rapidly	Indirect; no reported progression during limited follow-up	Very small sample and limited tumor follow-up	Phenotype-matched case signal.
Tocilizumab/Sarilumab	IL-6 pathway	Heterogeneous established irAEs	Established human toxicity; prevention/early concurrent animal treatment	31-patient melanoma cohort plus two tumor models (5)	Seventy-four percent improved after a median of 2 months	Direct but short-term in mice; indirect best-response comparison in humans	Uncontrolled heterogeneous cohort; prior best response may persist	Supportive human signal; durability unproven.
Special clinical context								
Sirolimus	mTOR modulation	Transplant rejection and colitis after PD-1 blockade	Established toxicity	Single kidney transplant recipient (28)	Graft tolerance and colitis control reported	Direct single-case observation of maintained tumor activity	Transplant context and multiple concomitant treatments	Non-generalizable single-case clue.

This table summarizes representative interventions. Intervention timing is reported as prevention/concurrent treatment, early treatment at toxicity onset, treatment of established toxicity, or rescue treatment for severe or life-threatening toxicity when the source permits that distinction. Antitumor outcomes are described as directly assessed, indirectly inferred, not assessed, or not adequately assessed when limited oncologic information was available. Direct assessment denotes a post-intervention tumor endpoint in the same cohort or model and does not by itself establish durable tumor control.

This table focuses on selected organ-directed, pathway-targeted, steroid-sparing, or rescue strategies discussed in the review and is not intended to summarize standard guideline-based management of all irAEs.

ICI, immune checkpoint inhibitor; irAE, immune-related adverse event; TRM, tissue-resident memory T cell.

## 5 Clinical studies must evaluate toxicity resolution and tumor control concurrently

### 5.1 Dual outcomes and time-related bias

Symptom improvement or glucocorticoid tapering alone cannot establish that a treatment preserves antitumor activity. Toxicity assessment should include clinical and objective organ responses, cumulative exposure to glucocorticoids and other immunosuppressants, and continuation or resumption of ICI therapy. Oncologic assessment should include radiographic response and its duration, progression-free survival, and overall survival. Particularly in patients who have already responded before immunosuppression begins, the prior best response cannot substitute for the subsequent duration of response or risk of progression. Retrospective studies comparing glucocorticoid monotherapy with additional immunosuppressants have incorporated both toxicity and tumor outcomes, but remain affected by treatment selection and residual confounding (29).

Serial tracking of functionally defined tumor-reactive clonotypes in blood and, when feasible, paired tumor samples can complement radiographic and survival outcomes. In a small tissue-based study of patients with non-small-cell lung cancer, clonotype mapping combined with neoantigen-specificity assays identified tumor-specific CD8 and CD4 T-cell clones that persisted in peripheral blood for years after checkpoint blockade (30). Persistence, expansion, or contraction of such clones after immunosuppression could provide a biological readout of antitumor immunity, but cannot substitute for response duration, progression-free survival, or overall survival.

An irAE is a time-varying event that develops after treatment begins. Patients who experience an irAE generally must survive and receive ICI therapy long enough to enter the exposed group; classifying patients as having or not having an irAE from treatment initiation therefore creates immortal-time bias. A cross-indication analysis of atezolizumab used time-varying covariates to address this problem and found that the association between irAEs and survival varied by severity and affected organ (2). Observational studies must also address confounding by tumor type, line of therapy, combination regimen, glucocorticoid dose, and ICI interruption.

### 5.2 Human mechanistic studies, clinical treatment cohorts, and animal models answer different questions

Human tissue studies and functional experiments can identify candidate pathogenic cells, antigens, and local pathways, but usually cannot compare treatment effects directly. Case series and observational treatment cohorts can demonstrate clinical feasibility but are vulnerable to case selection, treatment timing, and concomitant interventions. Tumor-bearing animals and organoids can test whether toxicity and tumor responses change in different directions, but preventive concurrent dosing is not equivalent to treatment of established organ injury, and short-term tumor volume is insufficient to infer durable immune control.

Preservation of antitumor activity is directly assessed only when a post-intervention tumor endpoint is measured in the same treated patients or animals. A previously achieved best response or the absence of reported progression provides only indirect evidence, whereas studies without interpretable post-intervention tumor outcomes do not assess tumor preservation. Direct measurement can still be inadequate: short-term tumor volume in mice, a single-patient observation, or an uncontrolled before-and-after comparison leaves durable antitumor control unresolved.

Studies of MLCK1, IL-6, and NLRP3 all assessed toxicity and tumor control, but they addressed different questions. The MLCK1 study primarily tested concurrent barrier protection; the IL-6 study combined an animal intervention with uncontrolled human cases; and the experiment treating established NLRP3-associated cardiac injury did not include simultaneous tumor implantation (5, 17, 18). Vedolizumab, IL-5-axis inhibition, and the abatacept-based combination have generated human treatment signals, but study size, controls, and oncologic follow-up remain inadequate (24, 26, 27). None of these studies establishes that the tested intervention preserves long-term antitumor activity.

### 5.3 Organ, severity, and treatment timing determine the therapeutic objective

Guidelines distinguish ICI continuation, temporary interruption, permanent discontinuation, and the intensity of immunosuppression according to the affected organ and severity, reflecting differences in the immediate risk and reversibility of individual irAEs (1). Colitis may involve TRM cells, other T-cell subsets, myeloid cells, and the epithelial barrier (14, 16, 17). Severe myocarditis requires concurrent recognition of skeletal muscle, respiratory muscle, and cardiac conduction system involvement (8, 27) whereas eosinophilic toxicity may be driven by IL-5-dependent effector cells (26). A single measure of systemic immune activation cannot substitute for organ-specific mechanistic phenotyping.

Early inflammation may be dominated by cellular recruitment and effector amplification, whereas established tissue injury also requires organ support and barrier repair. A preventive intervention exposes some patients who would never develop severe toxicity to an additional treatment, so the required evidence for tumor safety and overall safety is higher than in life-threatening rescue. During critical illness, prevention of irreversible injury takes priority. Once the patient is stable, clinicians can reassess immunosuppression tapering, ICI rechallenge, or alternative antitumor treatment. Therapeutic use depends on the pathogenic mechanism, dose, timing, and treatment duration; describing a drug as selectively immunomodulatory does not define its clinical role unless toxicity benefit and longitudinal antitumor outcomes are evaluated together.

## 6 Clinical validation requires longitudinal tissue sampling, mechanism-enriched enrollment, and long-term dual outcomes

The first priority is longitudinal, paired measurement of tumor immunity and organ injury. Sampling before ICI therapy, at toxicity onset, immediately before mechanism-directed treatment, and during recovery can distinguish pretreatment susceptibility from active injury and persistent post-treatment changes. Peripheral blood should, where feasible, be paired with tumor, affected-organ, and unaffected control tissues. TCR and B-cell receptor tracking should be integrated with spatial localization, antigen identification, and functional assays so that tumor-reactive, tissue-pathogenic, and bystander clones are not conflated (9, 30).

The second priority is prospective treatment of established toxicity in mechanism-enriched cohorts. Enrollment should use a marker available when the clinical decision is made and should match the intervention to the dominant phenotype, such as eosinophilia for IL-5-axis blockade or colonic TRM/IFN-γ, myeloid, trafficking, or barrier signals for pathway-directed treatment (13, 14, 16, 17, 26). The critical test is a prospective comparison initiated after objective organ injury has been documented, with prespecified organ response, glucocorticoid exposure, ICI resumption, response duration, progression-free survival, and overall survival measured in the same patients.

The third priority is stage-appropriate validation with durable follow-up. More common colitis is suitable for mechanism-stratified prospective comparisons, whereas rare, rapidly fatal myocarditis may require multicenter prospective registries, shared core outcomes, and adaptive comparisons that evaluate pharmacodynamic treatment intensity alongside ventilatory, circulatory, and other organ support (27). Follow-up in both settings should capture infection, viral reactivation, delayed autoimmunity, ICI resumption, duration of tumor response, progression, survival, and long-term organ function. Only this concurrent follow-up can distinguish short-term anti-inflammatory activity from clinically meaningful selective immune modulation (**Figure 3**).

**Figure 3:**
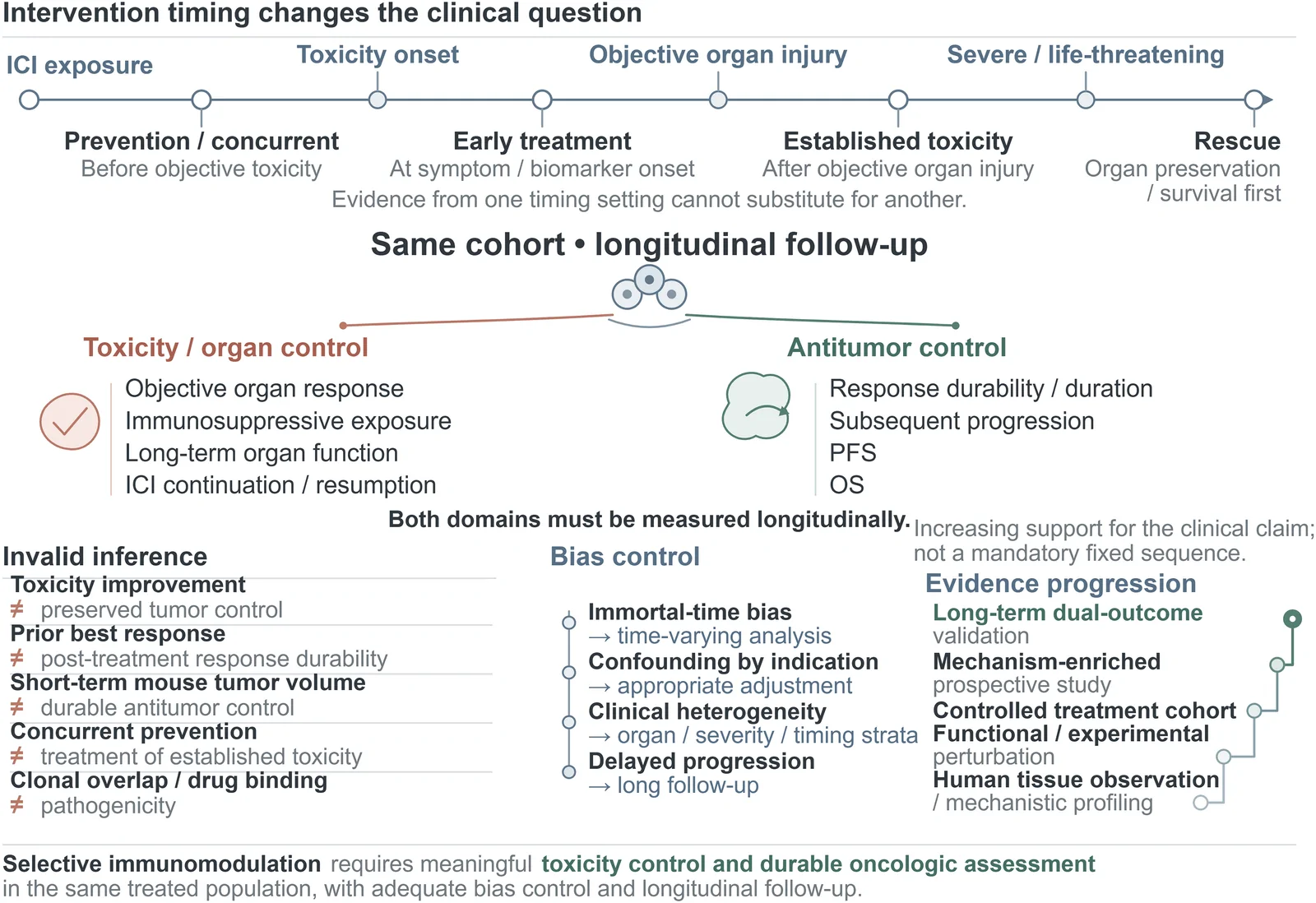
Evidence framework for evaluating selective immunomodulation of immune-related adverse events. Intervention timing determines the clinical question being tested. Prevention or concurrent treatment precedes objective toxicity, early treatment begins around symptom or biomarker onset, treatment of established toxicity follows documented organ injury, and rescue therapy during severe or life-threatening dysfunction prioritizes organ preservation and survival. Evidence from one timing setting cannot substitute for another. A clinically credible claim of selective immunomodulation requires longitudinal assessment of toxicity and oncologic outcomes in the same treated population. Toxicity assessment should include objective organ response, immunosuppressive exposure, long-term organ function, and ICI continuation or resumption when appropriate, whereas oncologic assessment should include response durability, subsequent progression, progression-free survival, and overall survival. Toxicity improvement, prior best tumor response, short-term tumor-volume changes, preventive treatment effects, clonal overlap, or drug binding should not be interpreted as stronger evidence than the corresponding study design supports. Observational studies additionally require attention to time-related bias, confounding, clinical heterogeneity, and adequate follow-up. Evidence supporting a claim of selective immunomodulation strengthens as mechanistic observations are linked to functional perturbation, treatment cohorts, mechanism-enriched prospective studies, and long-term dual-outcome validation; this progression is illustrative rather than a mandatory fixed sequence.

## 7 Conclusion

Antitumor activity and immune-related toxicity may share the release of immune tolerance, clonal expansion, and T–B-cell cooperation. Shared antigens and T-cell clones found across tumors and affected tissues provide mechanistic clues for the coexistence of efficacy and toxicity in some patients (7, 8, 11). After clones enter tissues, their function is further shaped by local antigen presentation and residency programs, which can themselves support antitumor immunity (4). In colitis, spatial cellular niches and epithelial barrier responses, and in selected cardiac models, innate inflammation, can sustain or amplify injury (16–18). Smaller tissue or fluid studies in other organs identify candidate local states but have not established a shared causal persistence program (19–21, 23).

Early signals have emerged for restricting organ homing, matching cytokine blockade to toxicity phenotypes, protecting tissue barriers, and using pharmacodynamically guided treatment during critical illness. Most findings, however, still come from case series, nonrandomized cohorts, or animal models. No strategy has yet been shown, after adequate control of bias and sufficiently long follow-up, to broadly reduce irAEs without compromising antitumor benefit. Future studies must evaluate organ recovery, immunosuppressive exposure, ICI resumption, and duration of tumor response concurrently within the same population.
